# Safety Comes First: Novel Styrene Butadiene Rubber (SBR) and Ethylene Propylene Diene Monomer (EPDM) Surfaces as a Response to Sport Injuries

**DOI:** 10.3390/ma14133737

**Published:** 2021-07-03

**Authors:** Cezary Strąk, Marcin Małek, Mateusz Jackowski, Ewa Sudoł

**Affiliations:** 1 Construction Materials Engineering Department, Instytut Techniki Budowlanej, ul. Filtrowa 1, 00-611 Warsaw, Poland; c.strak@itb.pl (C.S.); e.sudol@itb.pl (E.S.); 2 Faculty of Civil Engineering and Geodesy, Military University of Technology, ul. Gen. Sylwestra Kaliskiego 2, 00-908 Warsaw, Poland; marcin.malek@wat.edu.pl

**Keywords:** sports injuries prevention, sport surface, safety, rubber, EPDM, SBR

## Abstract

An athlete’s performance depends not only on the shoes they wear but also on the surface used in sports facilities. In addition, it can significantly contribute to reducing injuries, which are easy to get during sports competitions. In the present study, we wanted to investigate whether recycled styrene butadiene rubber (SBR) and ethylene propylene diene monomer (EPDM) could be used in the production of sports surfaces. For this purpose, we designed three different sports surfaces: (1) SBR covered with a thin EPDM spray layer on the top, (2) clean EPDM, and (3) bottom SBR layer with the top layer of EPDM. The test program of these surfaces included in its scope: shock absorption, vertical deformation, tensile strength, abrasion resistance, and slip resistance tests. Our research also involved the influence of the substrate under surface, temperature, and surface conditions. Presented results show that both materials, in the right proportions, can be used in the production of sports surfaces.

## 1. Introduction

Over the years, sports have become an indispensable part of human life, no matter if it is actively practiced or passively watched on television. This trend, however, resulted in a sharp increase in injuries caused by sports accidents taking place during physical activities [1,2]. Particularly popular injuries that athletes suffer are injuries to the limbs, including the feet and ankles, with a wide range of mechanisms: light, sharp, and even a long-term or complete disability [3,4]. Many of these injuries can be prevented by appropriate equipment, protective clothing, and safe sports surfaces providing adequate shock absorption after a fall.

However, specially designed sport surfaces can not only counteract injuries but also affect the results of athletes [5,6]. For this purpose, it is, of course, necessary to correctly select the component materials that will ensure, for example, adequate adhesion of the footwear to the ground and reduce the possibility of skidding [7]. The final material, which can meet the desired properties depending on the nature of the sport, is most often a combination of several different materials, e.g., polymers and composites. These mixes especially found a place in exposed pitches as they are constantly exposed to changing weather conditions. Polymer materials and composites, when properly designed, can withstand temperature fluctuations and the accompanying surface contractions, as well as the high humidity environment in the fall/spring period [8,9]. That may be a reason why recent years showed increased interest in these materials in the sport surface industry, as they are also assessed based on their functionality, strength, and water resistance. Moreover, they are doing remarkably well compared to traditional sport surface materials, such as asphalt or wood, which are way more expensive than polymer or composite materials.

One of the most suitable polymer materials for sport surfaces is ethylene propylene diene monomer (EPDM), which is an elastomer cross-linked in the sulfur or peroxide vulcanization process [10]. It has great properties for reversible deformation under the influence of mechanical forces while maintaining its structure [11,12,13]. Furthermore, EPDM exhibits high elasticity at low temperature and high heat resistance, which are crucial when speaking about the application in sports surfaces, especially outdoor ones. This material has a great ability to accumulate energy and high internal friction, as well. Accordingly, in coatings made with EPDM, energy is dissipated as a result of damping during deformation of the rubber, and the mere application of a compressive or tensile load causes elastic deformation of the material [14,15,16,17]. The process is completely reversible. These phenomena give EPDM a decisive technological advantage in applications, such as surfaces of sports facilities. In addition, another great rubber material for practical use in these is styrene butadiene rubber (SBR) as it has elasticity, frictional resistance, and mechanical strength similar to EPDM [18,19,20]. SBR is formed in the polymerization process, which allows for the production of a low reactive viscosity material with all the features of natural rubber, and was firstly developed in the 1930s by Interessen-Gemeinschaft Farbenindustrie AG in Germany [21,22,23]. Thanks to low costs, it has been used not only in everyday objects but also in protective coatings exposed to impact [24].

As all sport surfaces have direct contact with athletes, they must meet certain operational requirements to specify their safety. To do so, many aspects are taken into account, such as: shock absorption [25], vertical deformation [26], and slip resistance [27,28]. Moreover, sport surface should maintain its characteristics so that it can be used for years without visible breakage or abrasion. As presented by Kang and Lee [26], EPDM and SBR surfaces show promising results of reducing the impact after fall. Samples manufactured by them consisted one of four different types of rubber granules (three EPDM and one SBR) and one of six different types of one-component moisture-curing polyurethane resin. The highest force reduction reported by Kang and Lee was about 48% for the surface made from EPDM type C and the resin type D. All sports surfaces showed the vertical deformation in range of 1.1–2.0 mm and the tensile strength in range of 0.21–0.95 MPa. Furthermore, investigation of surface properties and elastomer behavior from EPDM/EOC/PP was also conducted by Uthaipan et al. [29]. They focused on the temperature influence on the tensile strength of surface and on the microscopic studies. The designed surface showed about 1 MPa tensile strength and evenly distributed grains in the composite matrix. Composites containing EPDM were also tested by Basak et al. [30]. They, however, focused on spectroscopic analysis, scanning electron and atomic force microscopy, and adhesion measurements.

Today, a growing tendency of waste and by-products usage in material production can be seen, e.g., polymers [31,32], composites [33,34,35,36,37], and even alloys [38]. This article aims to determine the properties of newly designed sports surfaces consisting of EPDM and SBR from recycling. Three different types of sport surfaces were tested, and the scope of the research involved the determination of properties, depending on the substrate used under surface, temperature, and surface conditions (wet or dry). So far, there is no knowledge about sports surfaces obtained from recycling materials’ behavior under specific weather conditions and on top of different substrates. Therefore, this study fills the information gap and presents the impact of underneath substrate used, temperature, and surface conditions on sport surface properties.

## 2. Materials

Sports surface slabs (4 pieces of each type) with dimensions of 1.500 m × 1.500 m × 0.015 m and the following structure, shown in Table 1, were prepared. Firstly, rubber granules (recycled EPDM and SBR or only recycled EDPM (Chełm, Poland)) and resin were mixed very carefully according to the designed weight proportions and purred in the mold. Then material was placed inside hot molding press and the 5 MPa pressure in the temperature of 150 °C was put on it. Samples then were cured in the laboratory conditions (22 ± 1 °C temperature and 60 ± 5% humidity) for 7 days. Then, the surfaces were cut into specimens with dimensions of 1.000 m × 1.000 m × 0.015 m for dynamic tests and of 0.150 m × 0.040 m × 0.015 m with a measurement part in the middle of 0.055 m × 0.025 m × 0.015 m for tensile strength tests.

## 3. Methodology

### 3.1. Tests Carried out Depending on the Substrate under Surface

**Figure 2:**
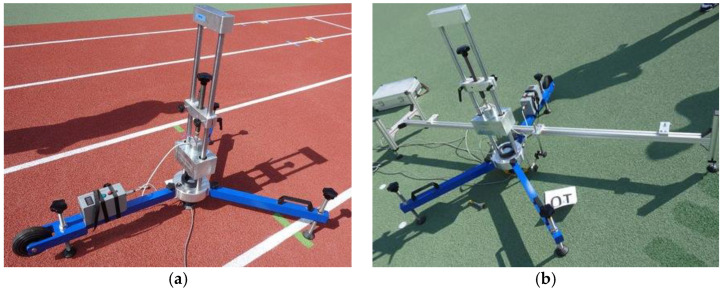
The artificial athlete used for test of (**a**) shock absorption, (**b**) vertical deformation.

The shock absorption and the vertical deformation tests were carried out in laboratory conditions according to EN 14808:2006 [39] and EN 14809:2005 [40], respectively. Both tests were conducted at three measuring points for each sample, and a device called an artificial athlete (Elektromechanika Marcin Sienicki, Warsaw, Poland) was used to perform them; see Figure 2. The shock absorption was expressed as the percentage reduction in force that a sports surface exerts compared to a hard concrete surface after the fall of device’s foot. Furthermore, the vertical deformation was described as the level of vertical displacement of the sports surface measured by a sensor (HBM, Poznan, Poland). The purpose of the tests was to determine the level of deflection and shock absorption of the sports surface as a result of running and jumping by the user, i.e., to express the degree to which the surface is set in motion during an impact.

### 3.2. Tests Carried out Depending on the Thermal Interaction Preceding Them

**Figure 3:**
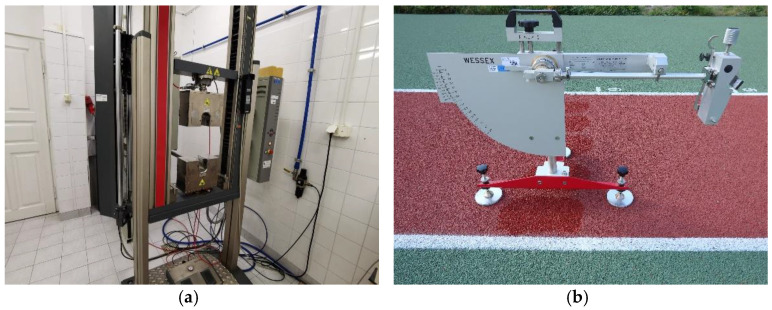
The apparatus used for the tests: (**a**) Zwick machine, (**b**) Taber apparatus.

**Figure 4:**
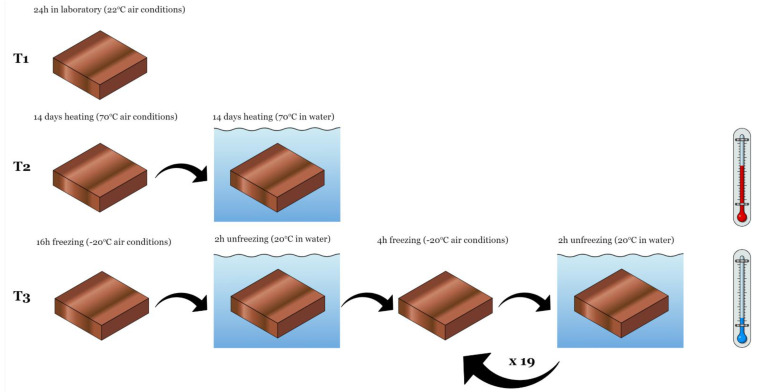
The scheme of thermal interactions preceding the tensile strength and the abrasion resistance tests: T1—conditioning in laboratory at 22 °C temperature for 24 h, T2—cycle of high temperature interaction, T3—cycle of freeze-thaw stresses.

The tensile strength test with the relative elongation at break and the abrasion resistance test were carried out in accordance with EN 12230: 2003 [41] and ISO 5470-1: 2016 [42], respectively. A Zwick machine (Zwick, Ulm, Germany) with a force range up to 10,000 N was used to test the tensile strength; see Figure 3a. The specimen was clamped in the jaws of the machine and then subjected to an axial tension until breakage. The result of the destructive force was recorded as the mean value of six measurements of appropriately prepared samples. The abrasion resistance test was carried out using the H18 type abrasive wheel, called Taber apparatus (TABER Industries, North Tonawanda, NY, USA), with a load of 1000 g rotating at a speed of 60 rpm; see Figure 3b. The number of conducted cycles was 1000. Both tests were performed for samples subjected to temperatures of +70 °C, +22 °C, and −20 °C. The scheme of the thermal interactions preceding the tests is shown in Figure 4.

### 3.3. Tests Carried out Depending on the Surface Conditions

The slip resistance of the sport surface was determined in accordance with EN 13036-4: 2011 [43]. In order to carry out the test, a British pendulum (WESSEX, Aldershot, England) was used with a CEN type 57 slipper, 76.2 mm wide and 126 mm long, and 55 IRHD rubber hardness. Before testing, the device was calibrated with reference surfaces (glass, reference plate, and polishing paper). The friction force between the shoe and the surface was determined by measuring the deflection of the pendulum during the movement of the shoe, using a calibrated scale. The C scale was used [43]. Tests of each sample were carried out at 3 measuring points for wet and dry surfaces at the temperature of 22 °C.

## 4. Results and Discussion

**Figure 5:**
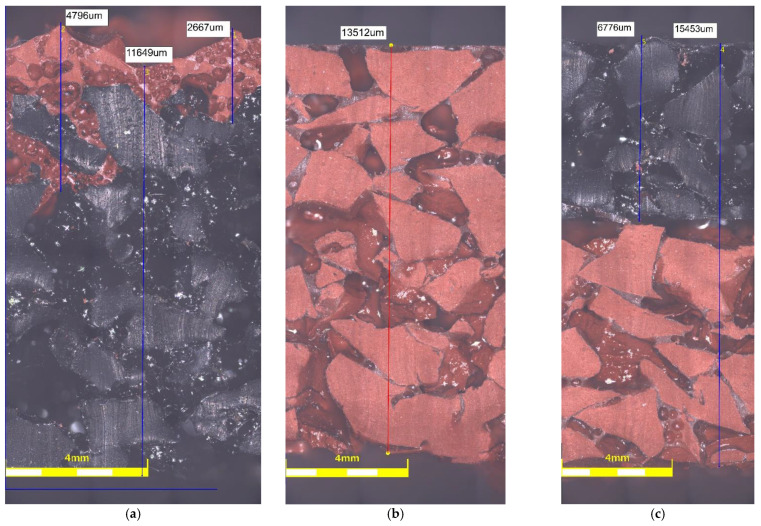
Light microscopy images of: (**a**) SBR covered with a thin EPDM spray layer on the top; (**b**) clean EPDM; (**c**) bottom SBR layer with the top layer of EPDM.

**Figure 6:**
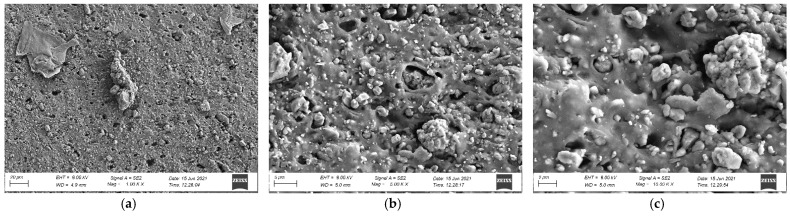

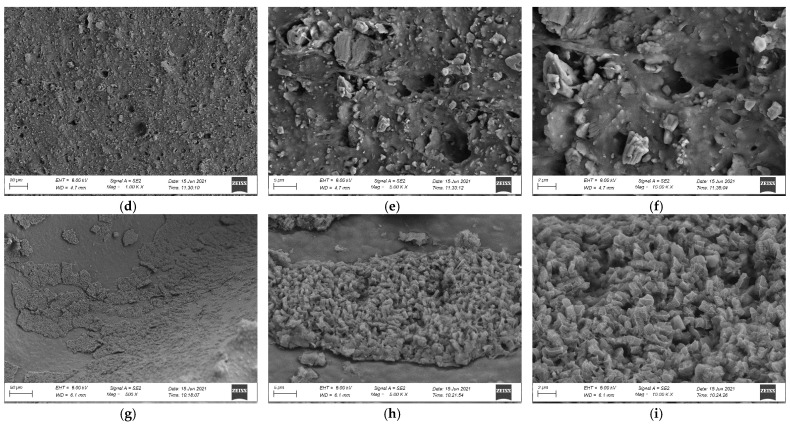
SEM photos of: SBR (**a**) 1.00 k magnification, (**b**) 5.00 k magnification, (**c**) 10.00 k magnification; EPDM (**d**) 1.00 k magnification, (**e**) 5.00 k magnification, (**f**) 10.00 k magnification; connection between SBR and EPDM (**g**) 0.50 k magnification; (**h**) 5.00 k magnification, (**i**) 10.00 k magnification.

The influence of sample components external structure is not apparent in tests; however, it may affect results of tests that are carried out. To get a better understanding of this, preliminary investigations of the microstructure of the tested sports surfaces were carried out on a stereoscopic microscope (DELTA OPTICAL, Nowe Osiny, Poland) enabling observation at a magnification of 8 to 80 times. The surface of each component grain is rough and clearly visible, as presented in Figure 4. In addition, all cross-sections show evenly distributed EPDM and SBR grains. In case of surface made as bottom SBR layer with the top layer of EPDM, there is a visible layer of polyurethane adhesive between them (Figure 5e,f) that is missing when it comes to SBR covered with a thin EPDM spray layer on the top samples. However, as shown in Figure 5a, the sprayed layer is highly porous and has good penetration into the SBR. In addition, the morphology of the cross-section surface was examined with a field-emission scanning electron microscope (SEM) Sigma 500 VP (Carl Zeiss Microscopy GmbH, Köln, Germany) that renders high-resolution images at low accelerating voltage. The samples were gold-coated before scanning to provide an electrically conductive surface. The accelerating voltage was 10 kV to avoid degradation of the sample. The observations were carried out at from 0.50 k to 10.00 k × magnification. The microstructures were observed on samples cut out perpendicularly to the surface. As presented in Figure 6a–I, all rubber samples show irregular surface and micro-voids in the internal structure of the recycled rubber that was not filled with the resin binder. This phenomenon has an impact on the roughness of the tested surfaces and their slip resistance, which are discussed later in the paper.

Studies of surfaces of EPDM rubber of three different hardness values have also been made by Mukhopadhyay [44]. The author used scanning electron microscope (SEM) to conduct his research and tried to find patter of failure mode of rubber parts. As he mentioned, microscopy technics are very important not only to predict the surface service life but also get a better inside of material structure and rubber grain distribution.

### 4.1. The Shock Absorption Test

Table 2 shows the shock absorption test results that are in the range of 33.1–52.4%. Similar range of shock absorption was reported by Khal and Lee [26] as they tested 24 different types of sport surfaces. Presented by them, test results were between 35% and 48%. The highest shock absorptions in our own study were reported for the SBR covered with a thin EPDM spray layer on the top surface, and the lowest shock absorptions were reported for the clean EPDM surface. In addition, the overall maintained trend for each sports surface was reported, regardless of its component materials and their proportions, as on a concrete substrate, the sports surfaces obtained the lowest shock absorption values. Compared to this type of substrate, the shock absorption values increased by about 3–4% and about 14–17% for the asphalt and mineral-rubber substrate, respectively, in every tested type of surface. On the basis of the conducted research, it was found that the mineral-rubber substrate allows for the greatest flexibility of the surface; thus, it is the most comfortable and safe to use. The high flexibility of the combined substrate and surface results in less stress on the joints and a lower risk of athletes’ injury after the fall.

**Figure 7:**
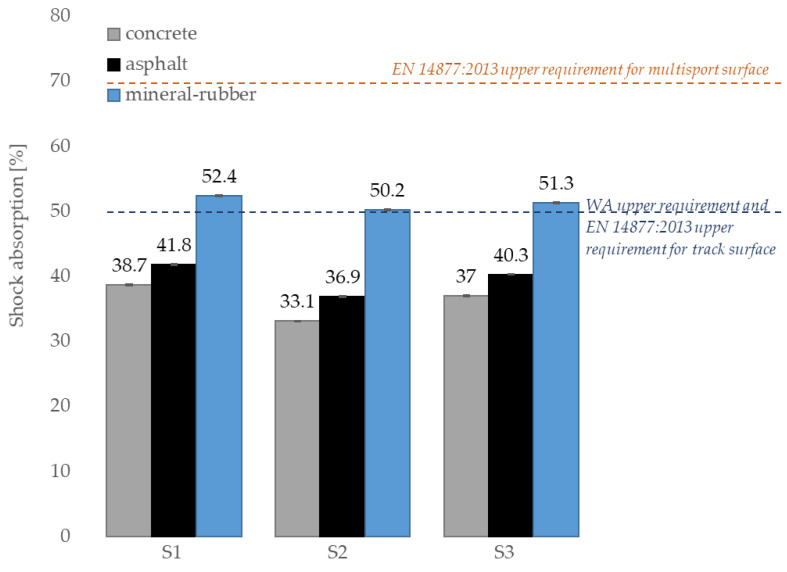
Shock absorption test results compared to EN 14877:2013 requirements and WA requirement.

Substrate made of a flexible mineral-rubber layer should be used only in multi-sport facilities, i.e., for practicing many sports (e.g., school playgrounds). The above statement is confirmed by comparing the obtained results with the World Athletics (WA) requirements [45] for the athletic facilities and the requirements of the EN 14877:2013 [46] standard. By analyzing them, it was found that the results of the shock absorption on the mineral-rubber substrate exceed the limits of the WA requirements and the requirements of EN 14877:2013 for track surface, but they fall within the requirements of EN 14877:2013 for multisport facilities. However, the results obtained on concrete and asphalt substrates meet the criteria set by both the WA and the EN 14877:2013 standard; see Figure 7.

### 4.2. The Vertical Deformation Test

**Figure 8:**
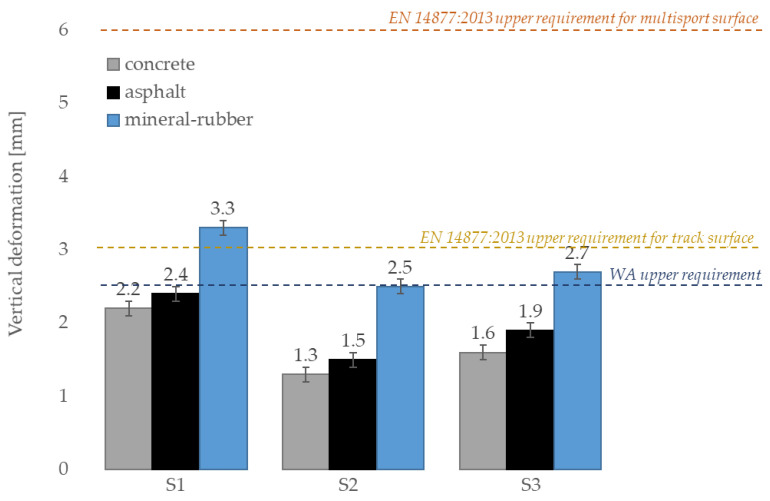
Vertical deformation test results compared to EN 14877:2013 requirements and WA requirement.

The vertical deformation of tested surfaces is presented in Table 2 depending on the substrate used under them. The highest deformation was reported for SBR covered with a thin EPDM spray layer on the top surface with the mineral-rubber substrate (3.3 mm) and the lowest deformation was reported for clean EPDM surface with the concrete substrate (1.3 mm). This shows a correlation between vertical deformation and shock absorption as the value distribution of both tests is identical. Furthermore, an increase in deformation was noted with the increase in flexibility of substrate used under surface. Compared to surfaces on top of the concrete substrate, surfaces on top of the asphalt and mineral-rubber substrates showed a higher vertical deformation of about 0.2–0.3 mm and 1.1–1.2, respectively. A similar conclusion was made by Yukawa et al. [47], who reported that the flexibility of the surface depends not only on the material but also on the substrate under it. In addition, athletes increase their leg stiffness (the stiffness of the integrated musculoskeletal system that behaves as a single linear spring during locomotion) when they are running on a compliant ground compared with running on a hard one, as Katkat [48] mentions. That is why surface should be tested taking into account the substrate beneath it, as well. Furthermore, from the point of view of the results achieved by competitive athletes, the most advantageous are surfaces installed on rigid substructures, i.e., concrete and asphalt. They allow obtaining better results than on elastic substrates made of mineral rubber materials. Therefore, according to the WA’s recommendations, track and runways should be installed on rigid substrates (Figure 8).

### 4.3. The Tensile Strength Test

**Figure 9:**
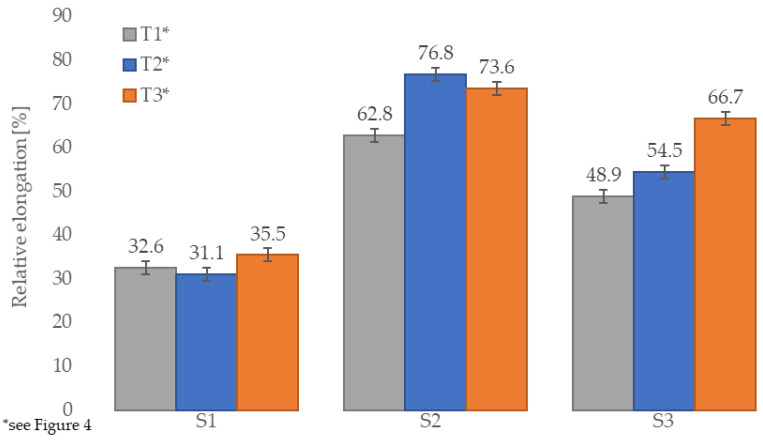
Relative elongation of samples depending on the thermal interaction preceding tensile strength test.

**Figure 10:**
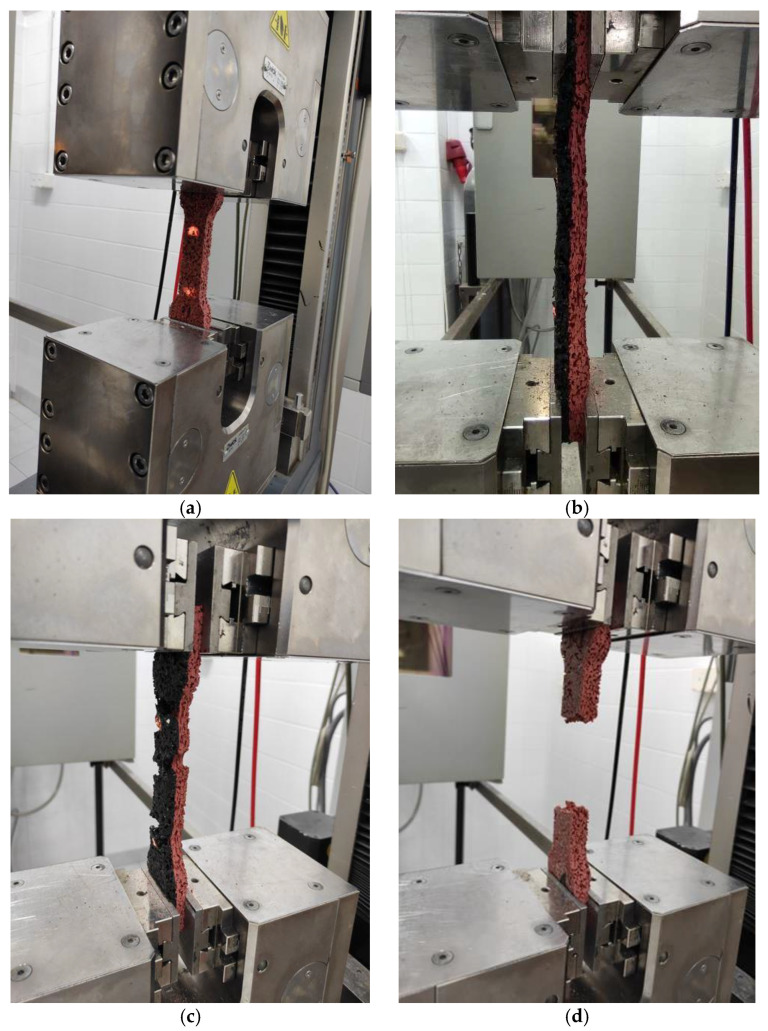
View of the sample during the static tensile strength test: (**a**) the sample mounted in the clamps, (**b**) the sample under load, (**c**) first cracks of the sample, (**d**) complete fracture of the sample.

**Figure 11:**
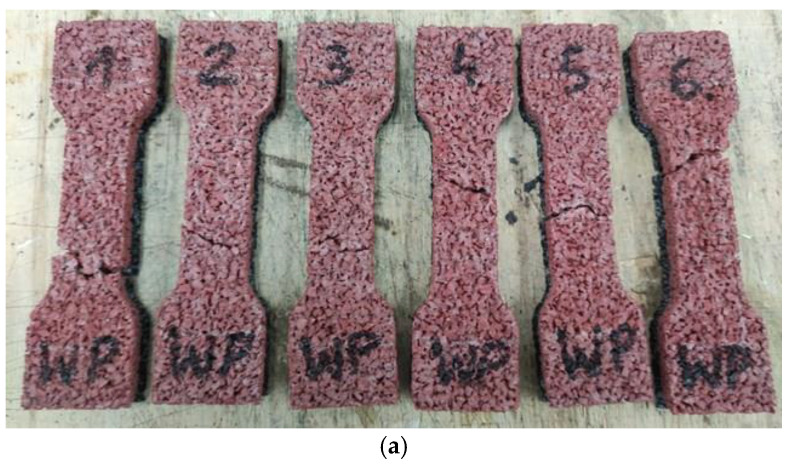

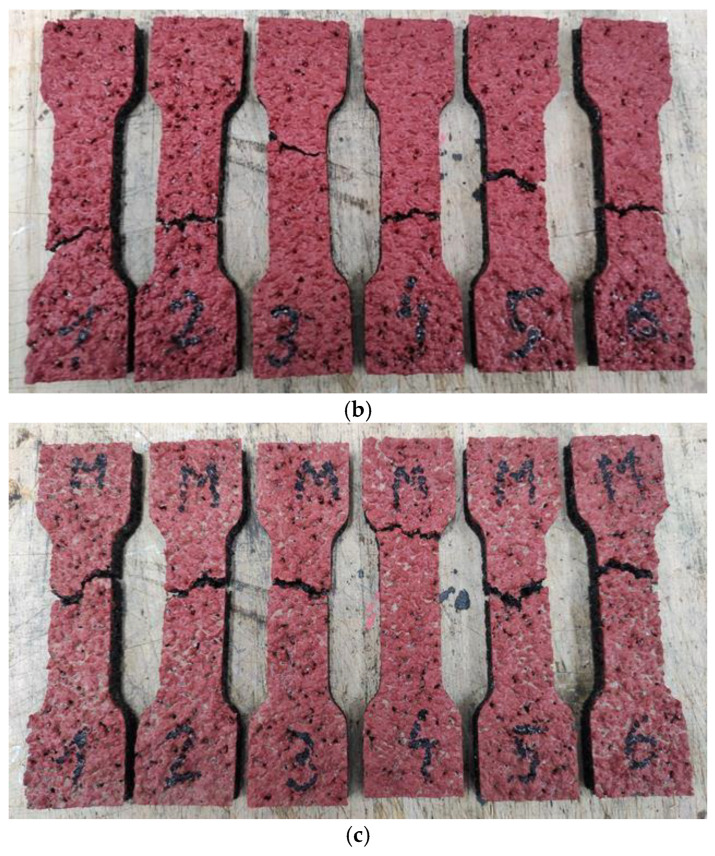
View of the samples after the static tensile strength test: (**a**) bottom SBR layer with the top layer of EPDM after high temperature interaction, (**b**) SBR covered with a thin EPDM spray layer on the top conditioned at 22 °C temperature, (**c**) SBR covered with a thin EPDM spray layer on the top conditioned at 22 °C temperature after the freeze-thaw stresses.

Analyzing the test results presented in Table 3, the highest values of tensile strength of sports surfaces were reported after the interaction of high temperature. The noted increases were about 37%, 26%, and 27% for S1, S2, and S3 samples, respectively, compared to the same samples conditioned at 22 °C temperature. The tensile strengths of the samples conditioned at 22 °C temperature and after the freeze-thaw stresses are similar in the case of S1 and S3 samples. This trend, however, is not relevant to samples fully made from EPDM because the values of S2 samples after freeze-thaw stresses were about 13% higher than the ones conditioned at 22 °C temperature. Furthermore, all tested materials showed high elasticity. The relative elongation values, measured during the tensile strength test, remained at a similar level for samples S1, regardless of whether the samples were subjected to thermal interaction before or just conditioned at 22 °C temperature; see Figure 9 and Figure 10. This was not reported, however, for samples S2 and S3 as a significant difference in their values was noted. The highest increase in relative elongation (17.8%) was for the bottom SBR layer with the top layer of EPDM surface samples conditioned at 22 °C temperature and samples after the interaction of high temperature. In addition, the peak value of relative elongation was reached by clean EPDM samples after freeze-thaw stresses. As shown in Figure 11, the breakpoint of the sample was difficult to predict due to the high porosity of the material and the random distribution of the rubber grains that differ in size. Only samples of SBR covered with a thin EPDM spray layer on the top after freeze-thaw stresses tend to break right in the middle. Overall, the pavement made entirely of EPDM has the best strength properties in every tested condition. In addition, no deterioration of the strength properties as a result of the applied thermal and humidity interactions was reported for each sport surface tested. Similar results of tensile strength were reported by Kang and Lee [26], as they tested EPDM and SBR sport surfaces. They reported the maximum value of this property for clean EPDM type C and resin type B surface (0.95 MPa), which was about equal to S2 samples’ tensile strength. Ethylene propylene diene monomer composite was also tested by Ismail and Mathialagan [49] and Mousa [50]. They reported about 2.2 times higher tensile strength (2.048 MPa and 2.000 MPa) compared to the clean EPDM tested in this study (S2 samples with 0.919 ± 0.05 MPa). This may be due to the additives used by Ismail and Mathialagan, and Mousa in the production of EPDM composites, as they both added zinc oxide, stearic acid, tetramethyl thiuram disulfide (TMTD), and 2-mercapto benzothiazole (MBT) to their composite.

Process of the tensile strength testing for all prepared samples proceeded the same way. In addition, all tested samples, regardless of the composition and content of individual components, showed high ductility during the tests; therefore, their destruction was not abrupt. First, the sample grew in length (Figure 10b). After reaching the maximum elongation, the first cracks appeared (Figure 10c), which gradually progressed, causing the complete rupture of the specimen; see Figure 10d.

### 4.4. The Abrasion Resistance Test

**Figure 12:**
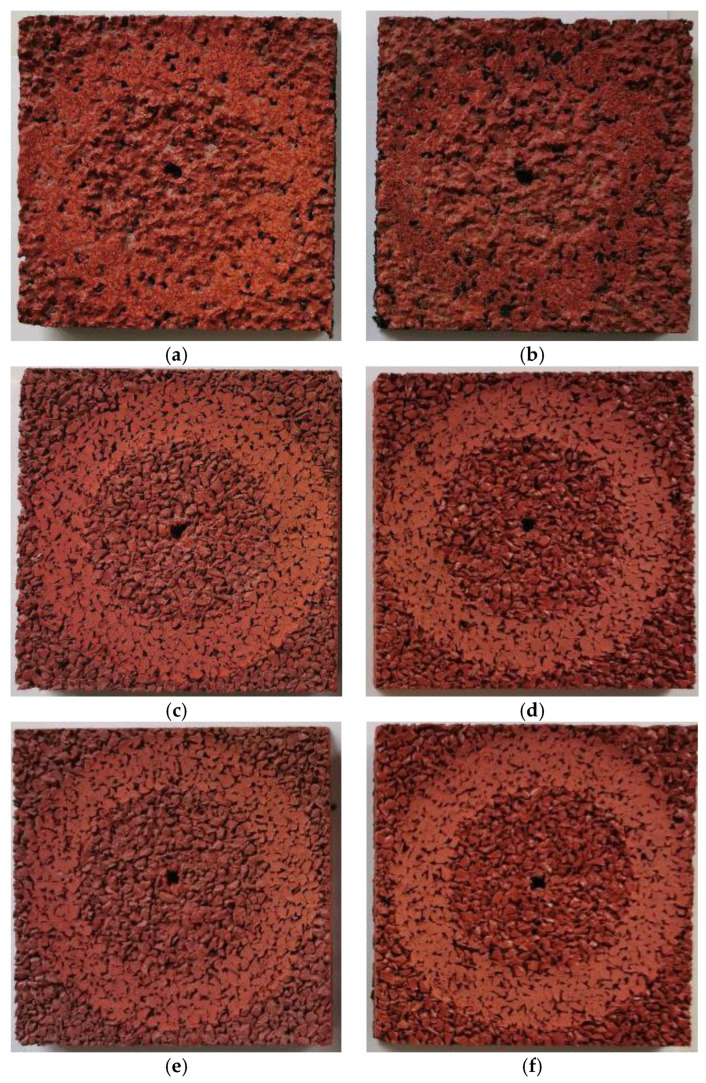
View of the samples after the abrasion resistance test: SBR covered with a thin EPDM spray layer on the top (**a**) conditioned at 22 °C temperature, (**b**) after the freeze-thaw stresses; clean EPDM (**c**) conditioned at 22 °C temperature, (**d**) after the freeze-thaw stresses; bottom SBR layer with the top layer of EPDM (**e**) conditioned at 22 °C temperature, (**f**) after the freeze-thaw stresses.

The results of weight loss due to abrasion of the surface shows Table 3. It was found that the impact of the thermal interactions is very insignificant, and the observed slight differences result from the variability of the surface as all values of the abrasion resistance test fluctuated for each surface type. The SBR covered with a thin EPDM spray layer on the top (S1 sample) showed the lowest weight loss, which indicates the highest abrasion resistance; see Figure 12. Other materials, clean EPDM and bottom SBR layer with the top layer of EPDM, showed slightly higher weight loss; however, it was still less than four grams, which is the boundary condition regarding weight loss due to abrasion according to EN 14877:2013 [46]. In addition, for S2 and S3 samples, a slight increase in weight loss was noted after freeze-thaw stresses and the interaction of high temperature. It may be related to the macroscopic degradation of the polymeric matrix which can be observed in terms of change in color and loss of gloss. This finding was reported by Wachtendorf et al. [51], as well, when they tested the influence of weathering on the leaching behavior of zinc and polycyclic aromatic hydrocarbons from synthetic sports surfaces.

### 4.5. The Slip Resistance Test

The slip resistance values of all surfaces, both wet and dry, are presented in Table 4. In wet conditions, the slip resistance values are at a similar level, and any differences result from the material variability and the measurement uncertainty. The best anti-slip properties among the 3 types of tested surfaces were reported for the SBR pavement covered with a thin EPDM spray layer (especially in dry conditions), marked as S1. This surface was characterized by the highest roughness (368 µm). Compared to samples S2 and S3, which had a roughness of about 320 and 231 µm, respectively, its roughness was about 15% and 59% greater. These results showed that the finish of the top layer is a determining factor in slip resistance. Analyzing the results in relation to the requirements of EN 14877:2013 [46], which are 55–110 PTV units in wet conditions and 80–110 PTV units in dry conditions), it was found that, in dry conditions, all the results were within the requirements, except for the average value of 111 PTV S1 samples. In wet conditions, the obtained values are at the lower limit of the requirements (55 PTV) or are just below this value. As reported bottom SBR layer with the top layer of EPDM surface, marked as S3, did not meet the requirements of the standard specifying outdoor sports surfaces in wet conditions [46].

## 5. Conclusions

In the present work, we aimed to clarify if recycled styrene butadiene rubber and ethylene propylene diene monomer could be used in sports surface manufacturing. This statement was confirmed by our own experimental studies, and, as obtained results show, all tested materials can be applied in sports facilities, either as track surface or multi-sport surface, depending on the substrate used under them. The obtained results of sport surfaces are as follows:

- SBR covered with a thin EPDM spray layer on the top surface showed the highest shock absorption, and clean EPDM surface showed the lowest shock absorption in every substrate tested.
- The vertical deformation was in range of 2.2–3.3 mm, 1.3–2.5 mm, and 1.6–2.7 mm for S1, S2, and S3 surface, respectively.
- The influence of the substrate on the shock absorption and vertical deformation was proven, as the values differ for the same type of surface depending on the substrate used.
- The highest tensile strength was reported for clean EPDM samples (0.919 ± 0.05–1.154 ± 0.05 MPa), and, compared to S1 and S3 samples, they showed about 2.2–2.6 and 1.5–1.8 times higher values, respectively.
- The influence of temperature on the tensile strength and abrasion resistance was reported, as obtained results vary for the same sample, depending on the temperature that it was conditioned in.
- The slip resistance of all tested surfaces were between 52 ± 1 and 56 ± 1 PTV in wet conditions and between 105 ± 1 and 111 ± 1 PTV in dry conditions, due to which the influence of surface conditions on slip resistance was proven.

## Figures and Tables

**Figure 1:**
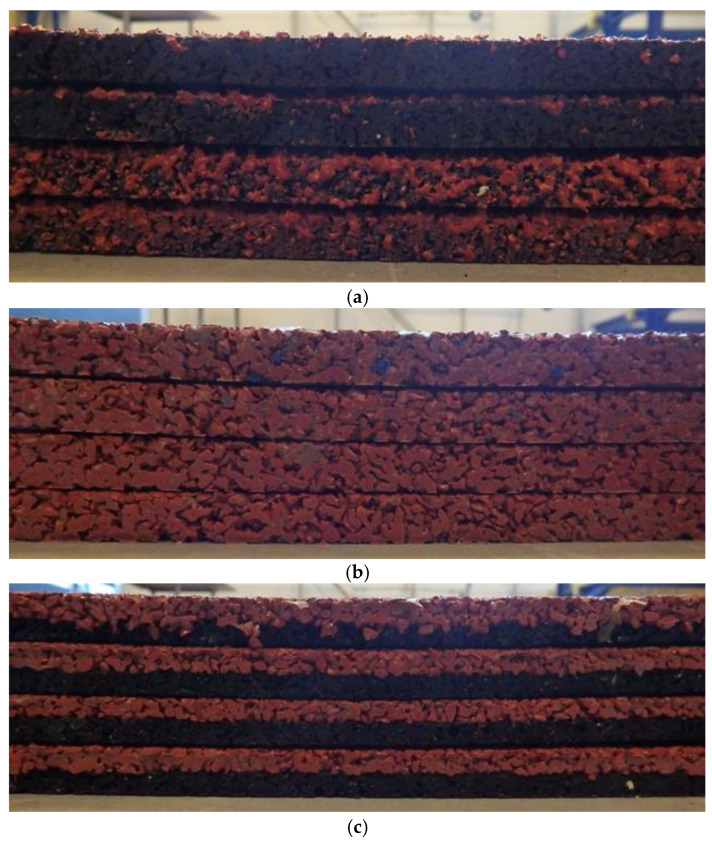
Digital images of the cross-section of the sports surfaces slabs prepared for the tests: (**a**) SBR covered with a thin EPDM spray layer on the top, (**b**) clean EPDM, (**c**) bottom SBR layer with the top layer of EPDM.

**Table 1:** Sample specification.

Surface Symbol	Description	Figure
S1	SBR covered with a thin EPDM spray layer on the top	Figure 1a
S2	clean EPDM	Figure 1b
S3	bottom SBR layer with the top layer of EPDM	Figure 1c

**Table 2:** Results of tests carried out depending on the substrate under surface (average values).

Surface Symbol	Shock Absorption (%)			Vertical Deformation (mm)		
	Concrete Substrate	Asphalt Substrate	Mineral-Rubber Substrate	Concrete Substrate	Asphalt Substrate	Mineral-Rubber Substrate
S1	38.7 ± 0.1	41.8 ± 0.1	52.4 ± 0.1	2.2 ± 0.1	2.4 ± 0.1	3.3 ± 0.1
S2	33.1 ± 0.1	36,9 ± 0.1	50.2 ± 0.1	1.3 ± 0.1	1.5 ± 0.1	2.5 ± 0.1
S3	37.0 ± 0.1	40.3 ± 0.1	51.3 ± 0.1	1.6 ± 0.1	1.9 ± 0.1	2.7 ± 0.1

**Table 3:** Results of tests carried out depending on the thermal interaction preceding them (average values).

Surface Symbol	Tensile Strength (MPa)			Abrasion Resistance (g)		
	T1 *	T2 *	T3 *	T1 *	T2 *	T3 *
S1	0.381 ± 0.03	0.395 ± 0.03	0.520 ± 0.04	0.728 ± 0.02	0.704 ± 0.02	0.726 ± 0.02
S2	0.919 ± 0.05	1.042 ± 0.05	1.154 ± 0.05	1.513 ± 0.03	1.412 ± 0.03	1.218 ± 0.03
S3	0.600 ± 0.04	0.570 ± 0.04	0.762 ± 0.04	1.423 ± 0.03	1.534 ± 0.03	1.431 ± 0.03

* see Figure 4.

**Table 4:** Experimental results of slip resistance (average values).

Surface Symbol	Slip Resistance Wet Samples (PTV)	Slip Resistance Dry Samples (PTV)
S1	56 ± 1	111 ± 1
S2	55 ± 1	106 ± 1
S3	52 ± 1	105 ± 1

## Data Availability

Data are contained within the article.
